# The influence of therapeutic hypothermia on the outcomes of cardiac arrest survivors: a retrospective cohort study

**DOI:** 10.3325/cmj.2020.61.40

**Published:** 2020-02

**Authors:** Marin Pavlov, Zdravko Babić, Ana Đuzel, Krešimir Crljenko, Mislav Nedić, Diana Delić Brkljačić

**Affiliations:** 1 Department of Cardiology, Sestre Milosrdnice University Hospital Center, Zagreb, Croatia; 2 University of Zagreb School of Medicine, Zagreb, Croatia

## Abstract

**Aim:**

To determine whether therapeutic hypothermia (TH) improves survival and neurological outcomes in out-of-hospital cardiac arrest (OHCA) survivors.

**Methods:**

This retrospective cohort study enrolled patients treated for OHCA with a return of spontaneous circulation admitted to the Cardiac Intensive Care Unit from October 2000 until March 2019. Data were collected from medical archives. Propensity score matching was used. The primary endpoint was death during hospital stay and secondary endpoint was cerebral performance category (CPC) score at discharge.

**Results:**

Out of 152 patients included in the study, 58 (38.7%) underwent TH treatment. After matching (which left 70 patients in the analysis), death during hospital stay occurred less often in TH group (28.6% vs 57.1%, *P* = 0.029), while the difference in CPC score was not significant. Cox proportional hazards model showed the predictors of death during hospital stay to be TH (hazard ratio [HR] 0.29, 95% confidence interval [CI] 0.13-0.68, *P* = 0.004), initial Glasgow Coma Scale score of 3 (HR 7.55, 95% CI 1.44-39.63, *P* = 0.017), and heart failure (HR 2.35, 95% CI 1.02-5.34, *P* = 0.045). TH was not an independent predictor of CPC score. Mann-Whitney U test and linear regression model showed that TH was associated with higher gain in GCS.

**Conclusion:**

TH was associated with better survival and certain variables suggesting improved neurological outcomes, suggesting that TH is a vital treatment option for comatose OHCA survivors.

In the last two decades, therapeutic hypothermia (TH) has gained critical appraisal as a method of improving survival and neurological outcome in comatose cardiac arrest survivors (1). Some of the possible mechanisms behind the beneficial role of TH are decreasing brain metabolism, lowering of intracranial pressure, diminishing brain cell apoptosis and necrosis, decreasing the release of lactate and excitotoxic compounds, reducing brain tissue inflammatory response and systemic inflammatory response syndrome, decreasing the production of free radicals, and limiting vascular and cell membrane permeability (2). Animal studies demonstrated a beneficial role of mild TH. For example, Safar et al (3) showed better overall performance, final neurological deficit score, and total brain histopathologic damage scores in dogs treated with mild TH (34°C) after 11 minutes of ventricular fibrillation as opposed to dogs that remained normothermic (37.5°C). Anecdotal experience from drowning accidents with circulatory arrest suggested promising results in humans (4). After a randomized control trial Hypothermia after Cardiac Arrest (HACA) (5) and a pseudo-randomized trial by Bernard et al (6), TH was introduced into routine daily practice. However, several subsequent studies presented ambiguous results (7-13). It is currently widely debated whether improved outcomes are attributable to the TH alone or to the whole bundle of care, consisting of optimal mechanical ventilation, analgosedation, urgent coronary angiography and intervention, infection control and treatment, and early rehabilitation. We hypothesized that, in a real-life setting, TH indeed improved the outcomes and represented an essential part of critical care for comatose cardiac arrest survivors. To test this hypothesis, we analyzed the data from 20 years of experience in treating such patients in our Center. We compared clinical, interventional, treatment, and outcome data of patients treated and not treated with TH.

## Patients and methods

### Study setting

The study was conducted at Sestre Milosrdnice University Hospital Center, a tertiary teaching center providing emergency care to an urban population of 350 000 inhabitants. Despite administrative territorial division, the hospital has a no-refusal policy for emergency patients, including patients with no valid insurance policy. The center’s autonomous cardiac intensive care unit (ICU) provides mechanical ventilation, renal replacement therapy, mechanical circulatory support, and interventional cardiology available 24/7.

Patients with out-of-hospital cardiac arrest (OHCA) are attended by mobile emergency units with at least one physician per unit, who is able to provide both basic and advanced life support. After a call, the closest emergency unit is dispatched to the scene. The resuscitation is performed by a team trained in emergency medicine according to international guidelines (14). On-field advance life support includes airway management with endotracheal tube or laryngeal mask in some cases, and recently more often with mechanical devices for chest compressions. Patients with return of spontaneous circulation (ROSC) are transferred to the emergency department (ED), where they undergo the initial work-up and consultation with an interventional cardiologist and intensivist. In patients with shockable initial rhythm, the decision whether to proceed with primary percutaneous coronary intervention (PCI) is made, often facilitated by urgent echocardiography. Patients with non-shockable initial rhythm in the ED often undergo additional work-up, mostly consisting of brain and pulmonary artery computed tomography and aortography, with the purpose to exclude other potentially treatable or non-cardiac causes of arrest.

### Patient data

All patients treated for OHCA with ROSC who were admitted to the cardiac ICU were eligible for enrollment. The inclusion criterion was cardiac arrest not witnessed by professional health care providers (ie, outside primary health physician’s office, ED, and ambulance). Patients who deceased in the ED were not considered for analysis. The analyzed time period was from October 2000 until March 2019. Patients were identified by reviewing medical histories, admittance protocols, and physicians’ and nurses’ shift change documentation. The latter was available in handwritten form for every shift until 2015. Since 2015, these data have been entered electronically in a local database. Every case with a diagnosis or remark suggesting cardiac arrest was reviewed. Upon case identification, all relevant clinical, work-up, interventional, treatment, and outcome details were collected from the medical archives. This included reviewing individual daily care, physical findings, vital parameters, medication lists, documentation describing patient behavior (compliance, ability to talk, eat, perform simple tasks, or walk with or without assistance), laboratory and radiographic work-up, and all interventional documentation, including video material. Overall, the data quality was acceptable. Out of 41 analyzed variables, 20 had no missing data and 16 had less than 5% missing data. The study was approved by the Sestre Milosrdnice University Hospital Center Ethics Committee (EP-13659/19-4).

### Therapeutic hypothermia

TH was introduced into routine practice at our institution in 2014 and since then has been offered to every eligible patient. The treatment was performed as follows: patients with ROSC and GCS<8 were considered eligible regardless of the clinical scenario (all patients were considered candidates, even in the absence of written consent, which was later obtained from family members). Analgosedation was performed using midazolam and fentanyl (since 2017 sufentanyl). All patients were intubated and mechanically ventilated. Core temperature was measured by esophageal temperature probe. TH was initiated in all patients within 6 hours from cardiac arrest. Rapid bolus (200-300 mL/min) of 2000 mL of cold saline (4°C) was given using pressurized bags via a large bore catheter inserted in the femoral vein. This was immediately followed by the administration of frozen ice-packs on large body areas. Additional 500 to 1000 mL of cold saline was allowed. The aim was to reach the target core temperature of 33°C (32-34°C) during 24 hours. For shivering, the use of additional boluses or infusion rate increments of analgosedation was allowed. If shivering was untreatable, vecuronium was used. Rewarming was passive, targeted at 0.5°C per hour. Upon reaching normothermia, analgosedation was withdrawn.

All patients were treated according to contemporary guidelines (14-16). Invasive treatment, including primary PCI, was strongly advocated before and regardless of TH.

The primary endpoint was death during hospital stay. Neurological disability was determined on arrival by Glasgow Coma Scale score (GCS), and at discharge by Cerebral Performance Category (CPC) score, which was the secondary endpoint (CPC 1 or 2).

### Statistical analysis

Categorical variables are presented as counts and frequencies, and the significance of differences between them was assessed with the χ^2^ or Fisher exact test. Continuous variables were tested for normality of distribution with the Kolmogorov-Smirnov test. They are presented as means and standard deviations (SD) or medians and interquartile ranges (IQR), and the significance of differences between them was assessed with the Mann-Whitney U test. Propensity score matching was performed with the following variables: age, GCS of 3 on admission, noradrenalin use, PCI, and treatment period since 2010. The missing values were excluded listwise, however, only GCS of 3 on admission had missing values (2.6%). Caliper was set to 0.05. Matching was performed without replacement. All analyses were performed on matched population (univariate analyses of unmatched population are presented in Table 1 for comparison). The quality of matching was assessed by a logistic regression model with TH as dependent variable and covariates of propensity score matching model. Cox proportional hazards model was used to determine the independent predictors of survival. Linear regression was used to determine the independent predictors of higher GCS gain. In both, Enter method was used. After matching, there were no missing data in variables included in the multivariable analyses. Multicollinearity was assessed by variance inflation factor (VIF). The mediation by admission year of TH’s effect on survival was assessed by using ordinary least squares and logistic regression path analysis modeling tool. Two-tailed significance tests were performed. *P* values lower than 0.05 were considered significant. All statistical analyses were performed in SPSS for Windows, version 25 (IBM SPSS, Armonk, NY, USA).

**Table 1:** Clinical, interventional, treatment, and outcome data of patients treated and not treated with therapeutic hypothermia (whole study population and matched population by means of propensity score matching)*

	Use of therapeutic hypothermia, n (%)^†^					
	whole population			matched population		
	no (n = 94)	yes (n = 58)	P	no (n = 35)	yes (n = 35)	P
Female patients	18 (19.1)	18 (31.0)	0.117	7 (20.0)	11 (31.4)	0.413
Age	61.4 ± 14.6	61.9 ± 13.7	0.869	61.9 ± 14.2	63.1 ± 14.9	0.755
Transferred patients^‡^	9 (9.6)	14 (24.1)	0.015	2 (5.7)	6 (17.1)	0.259
Diabetes mellitus	20 (21.7)	13 (22.8)	0.879	9 (25.7)	6 (17.1)	0.561
Hypertension	54 (59.3)	31 (54.4)	0.553	23 (67.7)	18 (52.9)	0.212
Active smoking	29 (31.5)	16 (28.5)	0.705	7 (21.2)	8 (23.5)	0.822
Previous myocardial infarction	23 (24.7)	12 (21.4)	0.645	7 (20.6)	6 (18.2)	1.000
Previous CABG or PCI	16 (17.2)	8 (14.5)	0.672	5 (14.7)	4 (12.1)	1.000
Cardiac arrest at/outside the home	36/55 (39.6/60.4)	25/33 (43.1/56.9)	0.668	10/24 (29.4/70.6)	12/23 (34.3/65/7)	0.797
Initial shockable rhythm	71 (82.6)	47 (81.0)	0.816	26 (86.7)	32 (91.4)	0.695
Witnessed arrest	52 (92.9)	51 (91.1)	0.728	16 (100.0)	32 (94.1)	1.000
Bystander CPR	27 (62.8)	27 (52.9)	0.336	7 (70.0)	18 (60.0)	0.715
Time until EMS	5.5 (5-10)	8 (5-10)	0.511	8 (5-10)	7 (4-10)	0.727
Time until ROSC	29.1 ± 15.9	29.3 ± 23.9	0.684	32.0 ± 19.2	28.3 ± 26.0	0.489
Use of mechanical chest compression devices	3 (3.1)	3 (5.2)	0.525	2 (5.7)	1 (2.9)	0.555
GCS on arrival	4 (3-15)	3 (3-3)	<0.001	3 (3-15)	3 (3-4)	0.331
Airway on arrival (tube/iGel)	52/11 (55.9/11.8)	28/17 (50.0/30.4)	0.013	17/9 (48.6/25.7)	16/11 (48.5/33.3)	0.734
Recurrent arrest	39 (42.4)	25 (43.1)	0.932	18 (51.4)	12 (34.3)	0.227
Mechanical ventilation	55 (59.8)	58 (100.0)	<0.001	25 (71.4)	35 (100.0)	0.001
Etiology of arrest:						
CMP	14 (14.9)	20 (34.5)	0.019	5 (14.3)	11 (31.4)	0.155
other	14 (14.9)	6 (10.3)		5 (14.3)	6 (17.1)	
ACS	66 (70.2)	32 (55.2)		25 (71.4)	18 (51.4)	
Urgent coronary angiography	38 (40.4)	45 (77.6)	<0.001	18 (51.4)	27 (77.1)	0.045
Urgent PCI	34 (36.2)	30 (51.7)	0.065	17 (48.6)	14 (40.0)	0.631
Analgosedation	4 (4.4)	58 (100.0)	<0.001	1 (2.9)	35 (100.0)	<0.001
Brain computed tomography	31 (33.7)	38 (66.7)	<0.001	15 (44.1)	23 (67.6)	0.087
Shock	26 (29.2)	27 (46.6)	0.032	15 (44.1)	10 (28.6)	0.216
Use of any vasopressor	17 (18.1)	25 (43.1)	0.001	10 (28.6)	8 (22.9)	0.785
Noradrenalin	8 (8.5)	25 (43.1)	<0.001	7 (20.0)	8 (22.9)	1.000
Inotrope (dobutamine)	20 (21.7)	26 (44.8)	0.004	11 (31.4)	12 (34.3)	1.000
LVEF	45 (35-55)	45 (30-55)	0.707	50 (40-59)	48 (30-55)	0.215
Heart failure	23 (27.1)	22 (37.9)	0.169	9 (25.7)	10 (28.6)	1.000
Pneumonia	23 (25.0)	29 (51.8)	0.001	10 (28.6)	18 (52.9)	0.051
Sepsis	6 (6.5)	14 (25.0)	0.001	2 (5.7)	9 (26.5)	0.023
Use of antibiotics	35 (38.5)	39 (69.6)	<0.001	15 (44.1)	26 (76.5)	0.013
Culture requiring isolation	3 (3.3)	12 (21.4)	<0.001	2 (5.9)	9 (26.5)	0.045
Tracheostomy	4 (4.3)	12 (20.7)	0.001	3 (8.6)	9 (25.7)	0.110
Acute renal failure	5 (5.4)	6 (10.5)	0.240	1 (2.9)	3 (8.8)	0.356
Acute renal replacement therapy	0 (0.0)	5 (8.6)	0.004	0 (0.0)	2 (5.7)	0.493
Length of hospital stay	7 (2-15)	12 (4-19.25)	0.019	5 (2-11)	15 (7-22)	0.001
Gain in GCS	0 (0-1)	2 (0-11)	<0.001	0 (0-0)	3 (1-12)	<0.001
CPC score 1 or 2 at discharge	47 (50.0)	22 (37.9)	0.147	12 (34.3)	17 (48.6)	0.332
Intrahospital mortality	40 (42.6)	23 (39.7)	0.738	20 (57.1)	10 (28.6)	0.029

*ACS – acute coronary syndrome; CABG – coronary artery by-pass surgery; CMP – cardiomyopathy; CPR – cardio-pulmonary resuscitation; CPC – cerebral performance category; EMS – emergency medical service; GCS – Glasgow Coma Scale score; LVEF – left ventricular ejection fraction; PCI – percutaneous coronary intervention; ROSC – return of spontaneous circulation; SD – standard deviation.

†mean ± standard deviation for normal distribution, median (interquartile range) for non-normal distribution.

‡patients transferred from other regional hospitals.

## Results

**Figure 1:**
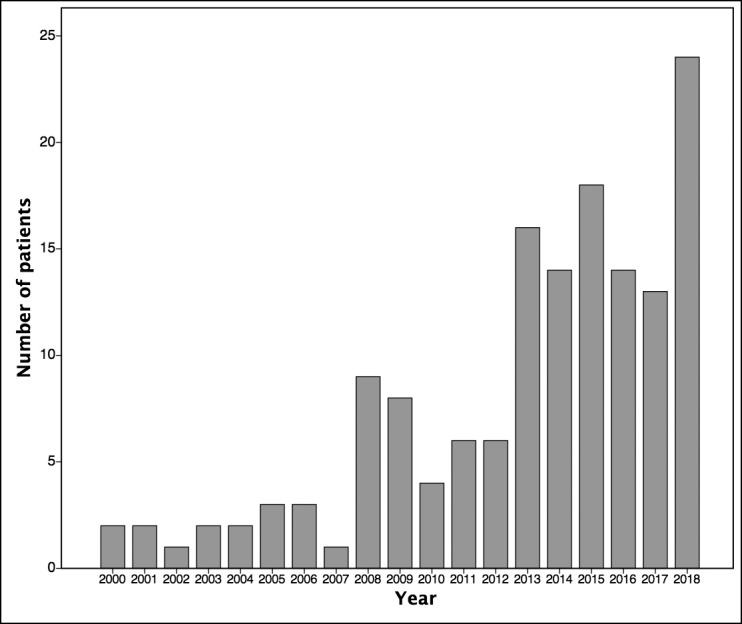
The number of patients admitted to the cardiac intensive care unit after out-of-hospital cardiac arrest with return of spontaneous circulation per year.

**Figure 2:**
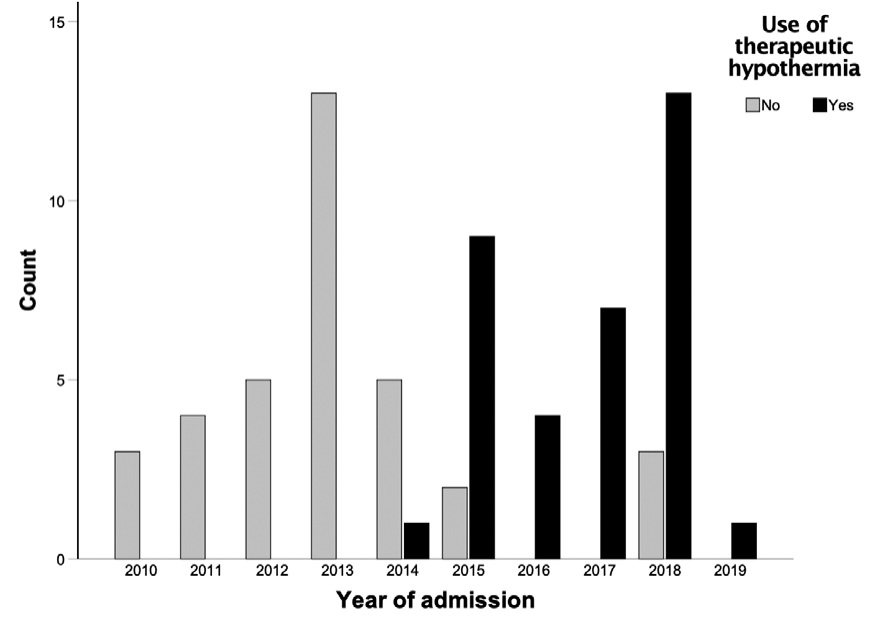
The number of patients after propensity score matching treated and not treated with therapeutic hypothermia according to the year of admission.

**Figure 3:**
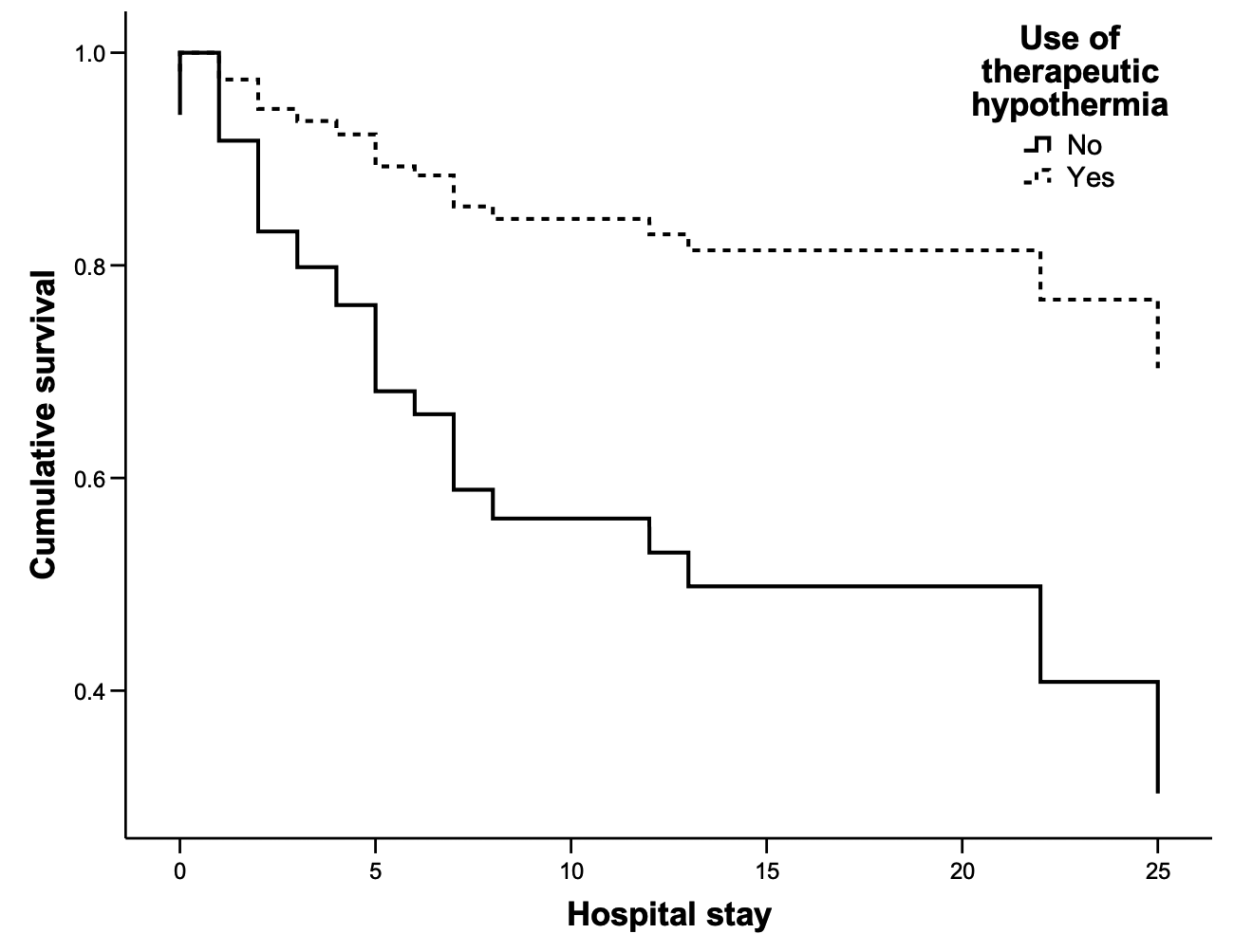
Cox proportional hazards model survival curves separated by the use of therapeutic hypothermia.

The study enrolled 152 patients. The admission trends are shown in Figure 1. The number of patients treated and not treated with TH according to the year of admission is shown in Figure 2. Out of all patients, 58 (38.7%) underwent TH treatment. The patients’ characteristics, clinical, procedure, and treatment details are presented in Table 1. After propensity score matching, 70 patients remained in the analysis (35 treated and 35 not treated with TH). In a logistic regression model created to test the quality of matching, pseudo R^2^ decreased from 0.467 before matching to 0.013 after matching. Univariate analysis is presented in Table 1. Death during hospital stay occurred less often in the TH group (*P* = 0.029), while there was no difference in CPC score at discharge (*P* = 0.332). A Cox proportional hazards model with eight covariates (age, sex, history of diabetes, PCI, TH, use of noradrenalin, initial GCS of 3, and heart failure) significantly predicted death during hospital stay (χ^2^ = 35.9, *P* < 0.001). Survival curves with separate lines for TH and no TH group are presented in Figure 3. Significant predictors of death during hospital stay were TH (hazard ratio [HR] 0.29, 95% confidence interval [CI] 0.13-0.68, *P* = 0.004), initial GCS of 3 (HR 7.55, 95% CI 1.44-39.63, *P* = 0.017), and heart failure (HR 2.35, 95% CI 1.02-5.34, *P* = 0.045). After adding variables that were significantly different between the TH and no TH group in univariate analysis (mechanical ventilation, urgent coronary angiography, use of antibiotics, sepsis) in the model, TH remained a significant predictor of survival (Table 2). The highest recorded VIF for a single covariate in multiple computations was 1.7. In mediation analysis, the indirect effect of TH on survival, as mediated by the year of admission, was not significant (effect -0.102, bootstrap 95% CI -1.50-1.15). Equal Cox proportional hazards model significantly predicted CPC score at discharge (χ^2^ = 22.2, *P* = 0.005). Significant predictors of CPC score at discharge were GCS of 3 on admission (HR 0.20, 95% CI 0.07-0.56, *P* = 0.002) and diabetes mellitus (HR 0.05, 95% CI 0.01-0.05, *P* = 0.008), but not the use of TH (HR 0.83, 95% CI 0.32-2.13, *P* = 0.696).

**Table 2:** Cox proportional hazards model assessing eight covariates in predicting survival. The model was significant with χ^2^ = 35.9 and *P* < 0.001*

	Significance	Hazard ratio	95% confidence interval for hazard ratio
GCS = 3 on admission	0.017	7.548	1.438-39.628
Therapeutic hypothermia	0.004	0.295	0.128-0.681
Heart failure	0.045	2.336	1.020-5.349
Age	0.162	1.024	0.991-1.057
Diabetes mellitus	0.188	1.915	0.728-5.041
Female patients	0.319	0.623	0.245-1.580
Noradrenalin	0.520	1.368	0.535-3.448
Primary PCI	0.632	1.278	0.469-3.482

*GCS – Glasgow coma scale score; PCI – percutaneous coronary intervention.

Neurological improvement was additionally expressed as the difference between the best and initial GCS score. The difference was significantly greater in patients treated with TH (*P* < 0.001). Linear regression was performed with variables used in the Cox proportional hazards models. Significant predictors of higher gain in GCS were TH (odds ratio [OR] 4.9, 95% CI 2.8-6.7, *P* < 0.001), noradrenalin use (OR -2.3, 95% CI -5.8 to -0.4, *P* = 0.027), and age (OR -2.1, 95% CI -0.2 to -0.002, *P* = 0.043). The highest VIF was 1.4, for age. VIF for the use of TH was 1.0.

## Discussion

This single-center observational cohort study showed that, after adjustments, TH was related to better survival and to certain variables suggesting improved neurological outcome at hospital discharge. The study population was predominantly male, with a high prevalence of patients with active smoking status and diabetes. The majority of arrests occurred outside the home. Despite the preponderance of witnessed arrests (9 out of 10 cases), lay-person resuscitation was performed in only 57.4% of patients. In a recent study from Ireland, bystander CPR was performed in as many as 84% of witnessed arrests (17). Riva et al (18) reported that the rate of OHCA with bystander CPR in Sweden increased from 30.8% in 2000-2005 to 68.2% in 2011-2017. Such results indicate the need for further efforts in the education of lay-person CPR on the national level in Croatia. It is currently accepted that providing bystander CPR doubles the survival rate in OHCA patients.

Emergency service response in our patients was prompt (median 7 min [5-10 min] for total study group), with 63.0% of all patients reached under the recommended 8 minutes (19). Further response time reduction in urban areas is exceptionally demanding, but rewarding. Burger et al (20) reported a 5% reduction in the probability of survival per 1-minute prolongation of emergency service response time. The mean time until ROSC of 29.2 ± 21.3 minutes in our study is similar to the times reported in other studies (21). Komatsu et al (22) reported this time to be 18.3 ± 15.1 minutes in patients with good neurological outcome (CPC score 1 or 2), as opposed to 48.6 ± 17.9 minutes in patients with worse CPC score or death. The respective times in our study were reasonably comparable, 16.9 ± 10.9 minutes and 40.8 ± 22.4 minutes. The most common cause of arrest was acute coronary syndrome, a fact directly related to a high rate of urgent coronary angiography and PCI.

The comparison of patients treated and not treated with TH was hindered by the differences between the two groups. Before the TH introduction, OHCA patients were treated less aggressively, no institutional protocols existed, and treatment options were less advanced. In such circumstances, the majority of the most severe patients did not reach the cardiac ICU, while those who did were in less critical condition. In addition, after TH introduction in 2014, the patients with poor GCS on arrival underwent TH, while the patients with substantial neurological recovery did not. Consequently, the TH group consisted of more patients with a worse initial GCS score; intubation on arrival; mechanical ventilation treatment; the diagnosis of shock, pneumonia, and sepsis; vasopressor, inotrope, and antibiotic treatment; and renal replacement therapy. To eliminate these differences, we performed propensity score matching. Currently, the only indication to withhold TH in OHCA patients is a good GCS on admission, which is why it was included in the matching. Besides GCS, other variables included in the matching are all known to be associated with the outcome. The greatest concern was controlling for the time of treatment. As since 2014 all patients with OHCA have been treated with TH unless they had good neurological status (only a small proportion), we were unable to adequately adjust the groups for the time variable. Eventually, we included the time variable “since 2010” in the matching process. This was chosen arbitrarily, as since 2010 no major infrastructural and staff change has occurred, and TH has been the most extensive treatment improvement. After matching, the majority of differences between TH and no TH group were balanced. The variables such as the use of analgosedation and mechanical ventilation, which are mandatory during TH, remained unbalanced. We were unable to prove that TH effect on survival was mediated by the year of admission. Several studies analyzed OHCA populations before and after TH implementation, with similar time frames. Van der Wall et al (23) reported no specific adjustment for the time of admission in a 10-year period. Sunde et al (24) compared patients treated and not treated with TH in two time periods (1996-1998 vs 2003-2005) and reported as possible confounders the improvements in chain-of-survival but performed no stratification by the time of treatment.

We found TH to be associated with better survival. Furthermore, Cox proportional hazards model analysis revealed TH as an independent predictor of survival. Similar results have been published earlier. Van der Wal et al (23) reported 20% relative risk reduction of intra-hospital mortality in OHCA survivors after TH. The reported mortality rates were higher than in our study (van der Wal: before/after TH 72.0%/65.4%; Pavlov: not treated/treated with TH 42.6%/39.7%). This can, at least partially, be ascribed to different protocols for OHCA patients (this study did not include OHCA patients without ROSC in ED; in the van der Wal study, all OHCA patients were directly admitted to the ICU).

In our study, TH was not associated with better neurological outcome expressed as CPC score at discharge in Cox proportional hazards model. Such finding can be explained by sample size and residual between-group differences. As a surrogate marker for neurological improvement, we used the difference between the best and initial GCS score. In linear regression, TH was an independent predictor of greater difference (ie, better recovery). Our results, although from a small cohort, can be used in conjunction with results from larger studies demonstrating the benefit of TH in comatose OHCA survivors (23-27).

Several randomized studies raised doubts as to the exact role of TH in the outcomes of OHCA patients. Strategies such as early (intra-arrest) employment of TH (7-9), core temperature targeted to 33°C or 36°C (10), or management duration of 48 compared with 24 hours (11), led to no difference between the groups, while implementing TH for in-hospital cardiac arrest yielded even worse survival and neurological outcomes (12). Additionally, certain registry data also suggested a lack of a positive effect of TH. Martinell et al (13) failed to prove improved survival and neurological outcome in 871 comatose OHCA survivors after adjustments for multiple confounders. The favorable treatment consequences may be explained by the so called Hawthorne effect. It is a phenomenon of altered performance (ie, over-performance) resulting from the awareness of being observed, for example as a part of a study (28,29). Currently, Targeted Temperature Management study 2 (TTM2 study) is recruiting patients with randomization to cooling to 33°C or to avoiding hyperthermia only (maintaining core temperature <37.8°C) (30). The results of TTM2 study may elucidate the exact role of TH in the treatment of OHCA patients.

The penetration of TH in Croatia is still low. The number of ICUs providing TH increased from 9% in 2008 (31) to 38.2% in 2017 (32). Yet, TH treatment is available to a greater proportion of patients, as comatose OHCA survivors from additional 29.4% of ICUs are urgently transferred through a primary PCI network to the ICUs providing TH (33). One quarter of patients treated with TH were transferred from two county hospitals, both around 55 km far from our Center.

This study has several limitations. Despite the effort involved in case identification, low proportion of missing data, and statistical procedures such as matching and multivariable analyses, a retrospective design inevitably creates the limitations in terms of the number of available variables, certain level of selection bias, and inability to control the data acquisition quality. The fact that the study was performed at a single tertiary teaching center may limit the applicability of these results to institutions where different approaches and protocols for treatment of OHCA patients may exist. The time frame covered is substantially wide, raising the doubt about the effect of the available treatment options and protocols. Over time, recommendations for the treatment of OHCA patients, including numerous complications observed in this study, have evolved. However, these changes would have affected both groups of patients equally. Furthermore, we attempted to overcome this limitation by including the admission year in propensity score matching. Contemporary prognostication measures, such as neuron-specific enolase levels and somatosensory evoked potentials, were not available for the majority of patients not treated with TH. Regression and propensity score matching analysis were to some extent hindered by the sample size.

To conclude, this study showed that, after appropriate adjustments, TH was associated with better survival and certain variables suggesting an improved neurological outcome. According to these results and our opinion, TH continues to be a critical part of treatment of comatose OHCA survivors. TH implementation changed the focus of intensive care societies toward further improvements in resuscitation and post-arrest care. However, despite numerous both positive and negative results, the exact role of TH in such patients’ outcomes is yet to be determined.

## References

[1] Bonaventura J, Alan D, Vejvoda J, Honek J, Veselka J (2016). History and current use of mild therapeutic hypothermia after cardiac arrest.. Arch Med Sci.

[2] Cariou A, Payen J-F, Asehnoune K, Audibert G, Botte A, Brissaud O (2018). Targeted temperature management in the ICU: Guidelines from a French expert panel.. Anaesth Crit Care Pain Med.

[3] Safar P, Xiao F, Radovsky A, Tanigawa K, Ebmeyer U, Bircher N (1996). Improved cerebral resuscitation from cardiac arrest in dogs with mild hypothermia plus blood flow promotion.. Stroke.

[4] Wanscher M, Agersnap L, Ravn J, Yndgaard S, Nielsen JF, Danielsen ER (2012). Outcome of accidental hypothermia with or without circulatory arrest: experience from the Danish Praesto Fjord boating accident.. Resuscitation.

[5] (2002). Hypothermia after cardiac arrest study group. Mild therapeutic hypothermia to improve the neurologic outcome after cardiac arrest.. N Engl J Med.

[6] Bernard SA, Gray TW, Buist MD, Jones BM, Silvester W, Gutteridge G (2002). Treatment of comatose survivors of out-of-hospital cardiac arrest with induced hypothermia.. N Engl J Med.

[7] Nordberg P, Taccone FS, Truhlar A, Forsberg S, Hollenberg J, Jonsson M (2019). Effect of trans-nasal evaporative intra-arrest cooling on functional neurologic outcome in out-of-hospital cardiac arrest: the PRINCESS randomized clinical trial.. JAMA.

[8] Bernard SA, Smith K, Cameron P, Masci K, Taylor DM, Cooper DJ (2010). Induction of therapeutic hypothermia by paramedics after resuscitation from out-of-hospital ventricular fibrillation cardiac arrest: a randomized controlled trial.. Circulation.

[9] Castren M, Nordberg P, Svensson L, Taccone F, Vincent JL, Desruelles D (2010). Intra-arrest transnasal evaporative cooling: a randomized, prehospital, multicenter study (PRINCE: Pre-ROSC IntraNasal Cooling Effectiveness).. Circulation.

[10] Nielsen N, Wetterslev J, Cronberg T, Erlinge D, Gasche Y, Hassager C (2013). Targeted temperature management at 33 degrees C versus 36 degrees C after cardiac arrest.. N Engl J Med.

[11] Kirkegaard H, Soreide E, de Haas I, Pettila V, Taccone FS, Arus U (2017). Targeted temperature management for 48 vs 24 hours and neurologic outcome after out-of-hospital cardiac arrest: a randomized clinical trial.. JAMA.

[12] Chan PS, Berg RA, Tang Y, Curtis LH, Spertus JA (2016). Association between therapeutic hypothermia and survival after in-hospital cardiac arrest.. JAMA.

[13] Martinell L, Herlitz J, Karlsson T, Nielsen N, Rylander C (2017). Mild induced hypothermia and survival after out-of-hospital cardiac arrest.. Am J Emerg Med.

[14] Monsieurs KG, Nolan JP, Bossaert LL, Greif R, Maconochie IK, Nikolaou NI (2015). European Resuscitation Council guidelines for resuscitation 2015: Section 1. Executive summary.. Resuscitation.

[15] Ibanez B, James S, Agewall S, Antunes MJ, Bucciarelli-Ducci C, Bueno H (2018). ESC Guidelines for the management of acute myocardial infarction in patients presenting with ST-segment elevation. The Task Force for the management of acute myocardial infarction in patients presenting with ST-segment elevation of the European Society of Cardiology (ESC).. Eur Heart J.

[16] Ponikowski P, Voors AA, Anker SD, Bueno H, Cleland JG, Coats AJ (2016). 2016 ESC Guidelines for the diagnosis and treatment of acute and chronic heart failure: The Task Force for the diagnosis and treatment of acute and chronic heart failure of the European Society of Cardiology (ESC). Developed with the special contribution of the Heart Failure Association (HFA) of the ESC.. Eur J Heart Fail.

[17] Barry T, González A, Conroy N, Watters P, Masterson S, Rigby J (2018). Mapping the potential of community first responders to increase cardiac arrest survival. Open Heart.

[18] Riva G Ringh M Jonsson M Svensson L Herlitz J Claesson A Survival in out-of-hospital cardiac arrest after standard cardiopulmonary resuscitation or chest compressions only before arrival of emergency medical services: nationwide study during three guideline periods. Circulation 2019[Epub ahead of print] 10.1161/CIRCULATIONAHA.118.038179 30929457

[19] Blanchard IE, Doig CJ, Hagel BE, Anton AR, Zygun DA, Kortbeek JB (2012). Emergency medical services response time and mortality in an urban setting.. Prehosp Emerg Care.

[20] Burger A, Wnent J, Bohn A, Jantzen T, Brenner S, Lefering R (2018). The effect of ambulance response time on survival following out-of-hospital cardiac arrest.. Dtsch Arztebl Int.

[21] Sauter TC, Iten N, Schwab PR, Hautz WE, Ricklin ME, Exadaktylos AK (2017). Out-of-hospital cardiac arrests in Switzerland: Predictors for emergency department mortality in patients with ROSC or on-going CPR on admission to the emergency department.. PLoS One.

[22] Komatsu T, Kinoshita K, Sakurai A, Moriya T, Yamaguchi J, Sugita A (2014). Shorter time until return of spontaneous circulation is the only independent factor for a good neurological outcome in patients with postcardiac arrest syndrome.. Emerg Med J.

[23] van der Wal G, Brinkman S, Bisschops LLA, Hoedemaekers CW, van der Hoeven JG, de Lange DW (2011). Influence of mild therapeutic hypothermia after cardiac arrest on hospital mortality.. Crit Care Med.

[24] Sunde K, Pytte M, Jacobsen D, Mangschau A, Jensen LP, Smedsrud C (2007). Implementation of a standardised treatment protocol for post resuscitation care after out-of-hospital cardiac arrest.. Resuscitation.

[25] Benson-Cooper KA (2015). Therapeutic hypothermia is independently associated with favourable outcome after resuscitation from out-of-hospital cardiac arrest: a retrospective, observational cohort study.. N Z Med J.

[26] Vaahersalo J, Hiltunen P, Tiainen M, Oksanen T, Kaukonen KM, Kurola J (2013). Therapeutic hypothermia after out-of-hospital cardiac arrest in Finnish intensive care units: the FINNRESUSCI study.. Intensive Care Med.

[27] Bae KS, Shin SD, Ro YS, Song KJ, Lee EJ, Lee YJ (2015). The effect of mild therapeutic hypothermia on good neurological recovery after out-of-hospital cardiac arrest according to location of return of spontaneous circulation: a nationwide observational study.. Resuscitation.

[28] Campbell JP, Maxey VA, Watson WA (1995). Hawthorne effect: implications for prehospital research.. Ann Emerg Med.

[29] Oddo M, Schaller MD, Feihl F, Ribordy V, Liaudet L (2006). From evidence to clinical practice: effective implementation of therapeutic hypothermia to improve patient outcome after cardiac arrest.. Crit Care Med.

[30] Dankiewicz J, Cronberg T, Lilja G, Jakobsen JC, Bělohlávek J, Callaway C (2019). Targeted hypothermia versus targeted Normothermia after out-of-hospital cardiac arrest (TTM2). A randomized clinical trial – Rationale and design.. Am Heart J.

[31] Gornik I, Lukic E, Madžarac G, Gašparović V (2008). Nationwide survey of hypothermia after cardiac arrest in Croatia.. Resuscitation.

[32] Đuzel A, Pavlov M, Babic Z (2017). Cardiac intensive care unit organisation in an economically less developed European country.. Intensive Care Med.

[33] Nikolic Heitzler V, Babic Z, Milicic D, Bergovec M, Raguz M, Mirat J (2010). Results of the Croatian primary percutaneous coronary intervention network for patients with ST-segment elevation acute myocardial infarction.. Am J Cardiol.

